# Retrospective analysis of subclavian venous catheterization procedures performed with ultrasound guidance by anesthesiology residents in the intensive care unit

**DOI:** 10.3389/fmed.2025.1675624

**Published:** 2025-09-26

**Authors:** Adem Yalçınkaya, Abdullah Kahraman, Cahide Kahraman, Atakan Sezgi, Ezgi Güngördü, Oral Mentes, Güler Eraslan Doğanay, Melek Didik, Azra Ozanbarcı, Jülide Ergil

**Affiliations:** ^1^Department of Anesthesiology and Reanimation, Ankara Dr. Abdurrahman Yurtaslan Oncology Training Hospital, Ankara, Türkiye; ^2^Department of Anesthesiology and Reanimation, Ankara Bilkent City Hospital, Ankara, Türkiye; ^3^Department of Anesthesiology and Reanimation, Ankara Etlik City Hospital, Ankara, Türkiye; ^4^İntensive Care Medicine, Ankara Sanatorium Training and Research Hospital, Ankara, Türkiye; ^5^Department of Anesthesiology and Reanimation, Health Sciences University Ankara SanatoriumTraining and Research Hospital, Ankara, Türkiye

**Keywords:** ultrasound, central venous catheter, subclavian, anesthesiology, residency training

## Abstract

**Objectives:**

The aim of this study was to promote the safe implementation of subclavian catheterization under ultrasound guidance in the intensive care unit, a method associated with relatively low infection rates and enhanced patient comfort. Additionally, this approach seeks to encourage its prioritization by anesthesiology trainees and to increase its active utilization in anesthesiology training programs.

**Materials and methods:**

Following the approval of the Ethics Committee of Etlik City Hospital, confidence assessments were conducted before and after subclavian catheterization procedures performed under ultrasound guidance by anesthesiology trainees in the anesthesiology intensive care unit between 2023 and 2024. The procedures were then retrospectively analyzed, and complications were documented.

**Results:**

No major complications were observed in any of the patients undergoing the procedure in the intensive care unit. Retrospective analysis of 40 patients revealed that 23 (57.5%) were male and 17 (42.5%) were female, with a mean age of 73.4 ± 11.3 years. Thrombosis occurred in only one patient (2.5%). Confidence assessments conducted among anesthesiology trainees demonstrated a statistically significant increase in confidence levels following training. None of the patients developed pneumothorax or hemothorax.

**Conclusion:**

It was concluded that other catheterization methods (jugular, femoral) are more frequently preferred during anesthesiology residency training. Encouraging trainees to perform subclavian catheterization under ultrasound guidance could be more beneficial in managing critically ill patients during the specialization process.

## Introduction

Central venous catheterization involves the placement of a catheter into the femoral, jugular, or subclavian vein. It is a routine procedure performed by anesthesiologists. Each clinician often prefers a specific type of central venous catheterization, with some approaches carrying higher complication rates (1). Complications associated with subclavian catheterization include subclavian artery injury, hematoma leading to respiratory distress, hemothorax, pneumothorax, and cannulation failure (1, 2).

Traditionally, two methods are described for subclavian catheterization: the classical landmark technique and catheterization under ultrasound guidance (3). Although the landmark technique has been in use for many years, it is associated with a higher risk of pneumothorax and arterial injury. While this method can be used in ventilated intensive care patients, it has been argued that performing the procedure under ultrasound guidance may be safer to minimize risks (3, 4).

To increase the use of ultrasound-guided subclavian catheterization in intensive care, anesthesia trainees were trained by an expert experienced in this procedure and related academic studies. Training was conducted at the bedside in the intensive care unit with live, ultrasound-guided subclavian catheterization performed on patients with indications for central catheter placement. The selected trainees had prior experience with ultrasound-guided upper extremity blocks and were proficient in ultrasound use, needle handling, and eye-hand coordination.

The training included demonstrations of how to achieve optimal imaging, visualize the necessary structures, determine the appropriate angle for needle insertion, and execute the procedure effectively. Using a linear ultrasound probe, trainees were instructed to obtain the best transverse plane image under the clavicle for intervention. On the ultrasound screen, pleura, subclavian vein, and subclavian artery were visualized, and catheterization was completed by puncturing the vein with a needle under ultrasound guidance.

A single-question survey was administered to trainees before and after the catheterization training. The question, “How confident are you in performing this procedure?” was scored on a scale of 1–10 (1 being the lowest confidence, 10 being the highest). Responses were recorded separately before and after the training.

In this retrospective study, anesthesia trainees with prior experience in ultrasound-guided regional anesthesia were surveyed. After a live demonstration of the ultrasound-guided in-plane subclavian catheterization technique on one patient by an expert clinician, the trainees were provided with detailed instructions on the procedure’s steps. Subsequently, the trainees performed the catheterization procedure using the in-plane ultrasound-guided technique on 40 patients with various diagnoses in the intensive care unit.

During the procedures, patient demographic data, coagulation parameters, complications, and trainees’ confidence levels regarding the procedure were documented. Retrospective data on catheter usage duration were also collected.

## Materials and methods

During their intensive care training, anesthesia residents received training and practical application on ultrasound-guided subclavian catheterization. Afterward, residents completed a confidence survey on this topic. These data were recorded. The goal was to incorporate ultrasound-guided catheterization into anesthesia practice. Following this, the data were retrospectively reviewed after obtaining approval from the local ethics committee.

Patients who were followed in the intensive care unit and underwent ultrasound-guided subclavian catheterization during 2023–2024 were included in the study. Patients who used the classic landmark technique and underwent jugular and femoral vein catheterization were excluded from the study.

Anesthesia residents with previous hand-eye, needle-hand, and screen coordination were shown how to visualize the subclavian vein and subclavian artery using ultrasound. They were shown the angle of needle insertion for optimal visualization. Following this, an experienced intensive care specialist performed a live patient intervention. During the resident’s intervention, the experienced specialist accompanied and supported the procedure from beginning to end. In cases where the resident failed to complete the procedure, the experienced specialist completed the catheterization procedure. Cases taken over by the specialist were excluded from the study. Residents were given the right to perform a single procedure. In cases where the needle could not be visualized or repeated procedures were not permitted, and the procedure was completed by the experienced specialist. All procedures were performed by residents with 13–33 months of experience and active training in anesthesia. Only one procedure from each resident was included in the study.

All patients included in the study underwent subclavian catheterization using the in-plane technique under ultrasound guidance. The procedures were performed by anesthesia residents at different seniority levels under the supervision of an intensive care specialist. All procedures were performed with in-plane technique under ultrasound guidance. During the procedure, demographic data, coagulation parameters, and any complications in patients were documented. Additionally, the trainees’ confidence levels regarding the procedure post-training and the retrospective usage duration of the catheters were also recorded. A single-question survey was administered to the trainees before and after the catheterization training. The question was, “How confident are you in performing this procedure?”, and the trainees rated their confidence on a scale of 1–10 (1 = least confident, 10 = most confident). Responses were recorded separately before and after the training. After the procedure, all patients were evaluated with chest radiography to assess for pneumothorax and catheter malposition.

### Catheterization technique

**FIGURE 1:**
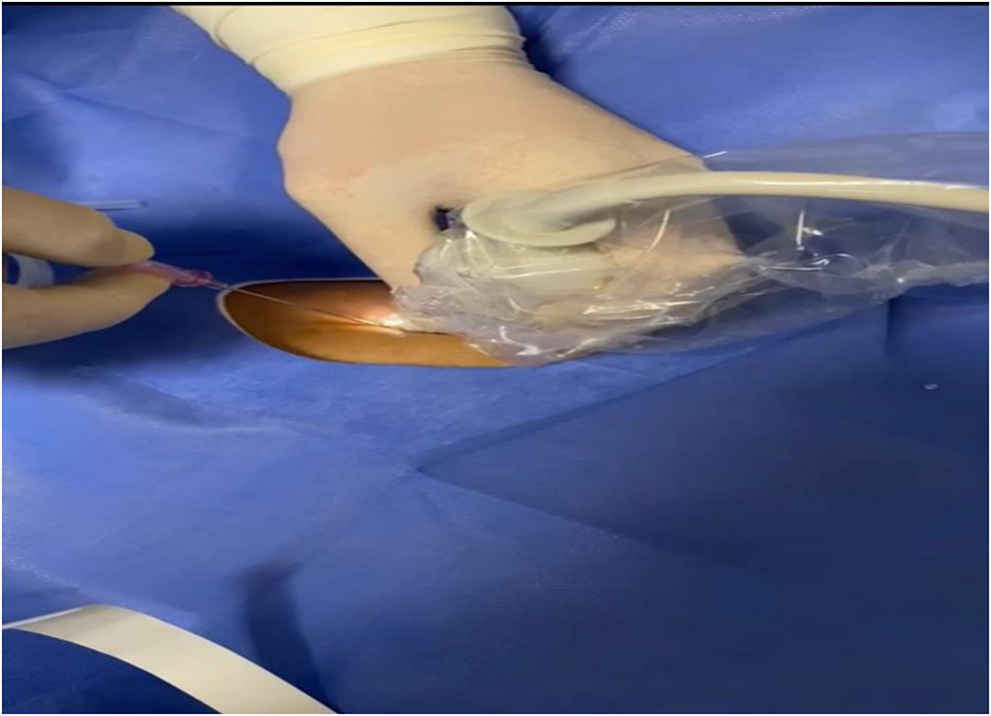
Needle direction.

Patients were placed in the supine position before the procedure. Using a 12 MHz linear ultrasound probe (Esaote Mylabsix SpA, Genoa, Italy), both the right and left subclavian veins and adjacent anatomical structures were examined. The side with the most optimal and highest-quality imaging was selected for the procedure (Figure 1).

After ensuring proper sterilization and sterile draping, 5 mL of 2% prilocaine diluted with 5 mL of 0.9% isotonic solution was injected into the infraclavicular region designated for the procedure. The subclavian vein, subclavian artery, and pleura were visualized in the longitudinal plane using ultrasound.

An 18G 6.5 cm introducer needle was advanced into the vein under ultrasound guidance, and blood aspiration confirmed venous access. A guidewire was then introduced into the subclavian vein through the needle. Finally, a three-lumen central venous catheter (Arrow International, Reading, PA, USA) was inserted into the subclavian vein using the Seldinger technique.

### Statistical analysis

The study data were analyzed using Jamovi version 2.6.0.0. The suitability of variables for normal distribution was examined using the Shapiro-Wilk test.

For continuous variables with normal distribution, Pearson correlation coefficients were used. For non-parametric data, relationships between numerical variables were analyzed using Spearman Rank correlation coefficients. Relationships between variables in independent groups were assessed using the Mann-Whitney U and Kruskal-Wallis tests (1, 2).

Descriptive statistics for numerical variables were presented as mean, median, and standard deviation. Categorical variables were expressed as frequencies and percentages *n*(%). A significance level of 0.05 was considered for all analyses.

## Results

The retrospective analysis of 40 patients who underwent subclavian catheterization under ultrasound guidance performed by anesthesiology trainees in the intensive care unit revealed the demographic data summarized in Table 1.

**TABLE 1:** Demographicand clinical characteristics of patients.

Variable	Number (%)	Mean ± SD
**Gender**		
Female	17 (42.5%)	–
Male	23 (57.5%)	–
Age	–	73.4 ± 11.3
Body mass index	–	25 ± 5.7
**Admission diagnoses**		
COPD	8 (20%)	–
Acute kidney injury	4 (10%)	–
Acute stroke	5 (12.5%)	–
Intracranial hemorrhage	2 (5%)	–
Lung malignancy	3 (7.5%)	–
Infective endocarditis	1 (2.5%)	–
Post-CPR	3 (7.5%)	–
Sepsis and septic shock	5 (12.5%)	–
Multi-trauma	4 (10%)	–
Coronary artery disease	3 (7.5%)	–
Motor neuron disease	2 (5%)	–

COPD, chronic obstructive pulmonary disease; CPR, cardiopulmonary resuscitation.

All 40 patients included in the study (100%) were on low-molecular-weight heparin (LMWH). Additionally, two patients were receiving both acetylsalicylic acid and LMWH, while one patient was on LMWH, acetylsalicylic acid, and clopidogrel. The average prothrombin time (PT) was 10.2 ± 1.86 s, the activated partial thromboplastin time (APTT) was 29.8 ± 3.29 s, and the international normalized ratio (INR) was 1.1 ± 0.14. The platelet count ranged from a minimum of 91 × 10^9^/L to a maximum of 257 ± 158 × 10^9^/L (Table 2).

**TABLE 2:** Coagulation parameters.

Parameter	Mean	Standard deviation
PT (sec)	10.24	1.86
aPTT (sec)	29.78	3.29
INR	1.14	0.14
Platelets (/μL)	257,600	158,201

Catheter malposition was evaluated using ultrasound during the procedure and confirmed with postero-anterior chest radiography after the procedure. No cases of catheter malposition were observed. Thrombosis developed in one patient on the third day following the procedure, leading to catheter removal and treatment with LMWH. This patient had no subsequent complications. Other complications such as arterial injury, bleeding, pneumothorax, hemothorax, or nerve damage were not observed in any of the patients.

The mean catheter usage duration was 20.5 ± 7.8 days. A moderate positive correlation was found between catheter duration and platelet count (Pearson’s *r* = 0.406, *p* = 0.009), but this was not considered clinically significant. In the literature, it has been shown that there is no statistical difference in the number of catheter days when patients with mild thrombocytopenia (PC: 100–150/nl), patients with moderate thrombocytopenia (PC: 50–100/nl), patients with severe thrombocytopenia (PC: <50/nl) are compared with patients with normal platelet count (5).

There is no significant relationship was found between the procedural success, as well as the number of attempts, and age, gender and BMI (*P* > 0.05)

In our study, none of the 40 patients who underwent ultrasound-guided in-plane subclavian catheterization performed by anesthesia trainees developed pneumothorax.

**FIGURE 2:**
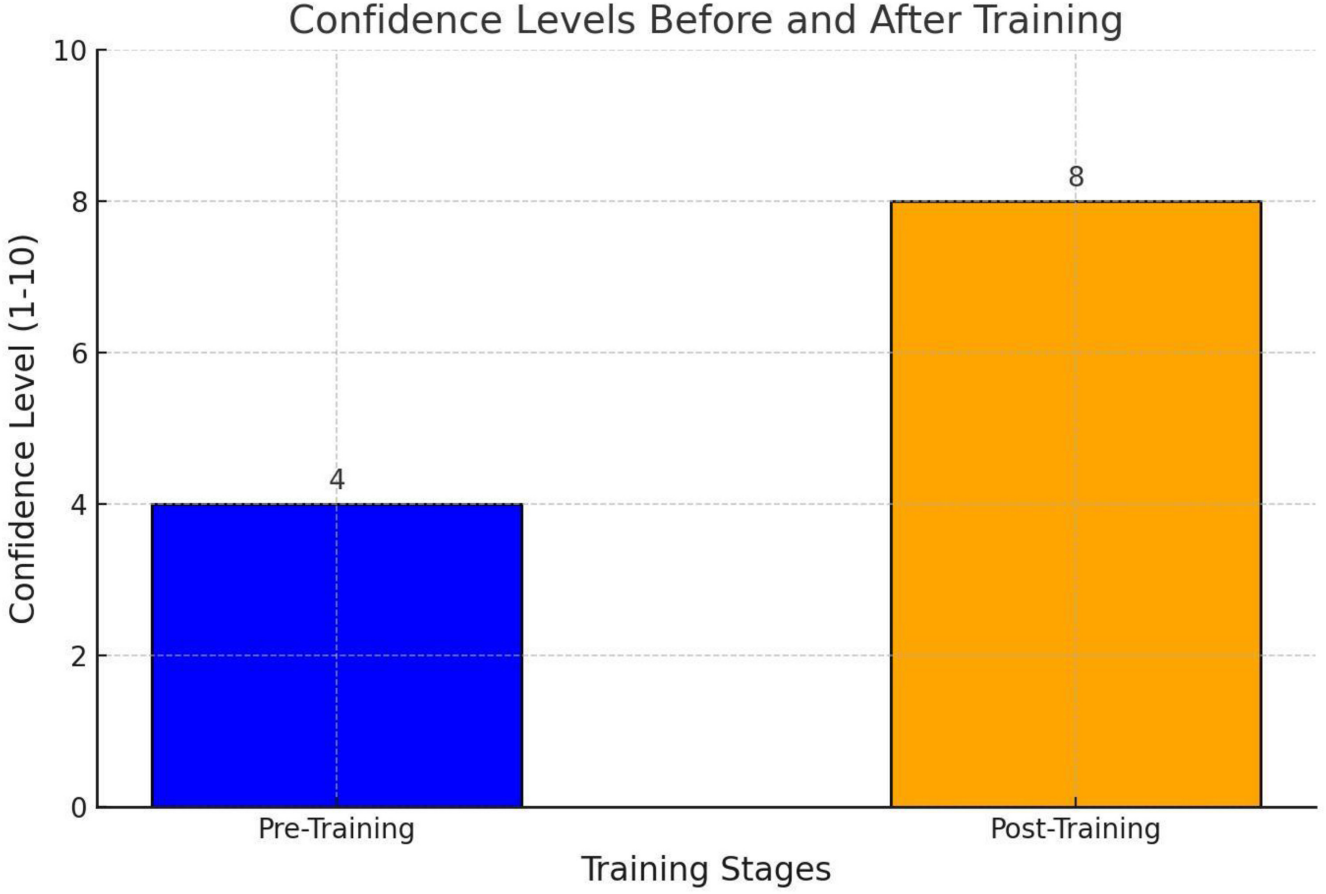
Self -confidence survey result.

Anesthesia trainees were assessed for their confidence in independently performing subclavian catheterization using a single-question survey, rated on a scale from 1 to 10 (1 = lowest confidence, 10 = highest confidence). The average confidence score increased significantly from 4 before training to 8 after training (Figure 2). This increase was statistically significant.

These findings indicate that ultrasound-guided subclavian catheterization is a safe procedure with minimal complications and significantly improves the confidence of anesthesia trainees in performing the technique. It should be noted that our study has limitations such as the small number of patients and being a single-center retrospective study

## Discussion

The use of real-time ultrasound has become routine in internal jugular and femoral vein catheterization, with numerous studies demonstrating its ability to reduce complication rates associated with the procedure (6). However, due to the challenges in obtaining clear imaging and the procedural difficulties involved, ultrasound has not yet become standard practice for subclavian catheterization (3, 7).

In the literature, the incidence of pneumothorax associated with subclavian catheterization is reported to be approximately 1%–3% (8). In our study, none of the 40 patients who underwent ultrasound-guided in-plane subclavian catheterization performed by anesthesia trainees developed pneumothorax. While previous studies have shown that the use of ultrasound reduces the risk of pneumothorax, it does not eliminate the risk entirely. The absence of pneumothorax in our study, despite the involvement of trainees inexperienced in subclavian catheterization, aligns with these findings. It should be noted that our study has limitations such as the small number of patients and being a single-center retrospective study.

Some studies using ultrasound report malposition rates as high as 10%, with the most common malposition being catheter entry into the ipsilateral internal jugular vein (9). In our study, catheter malposition was evaluated intra-procedurally with ultrasound, specifically checking the internal jugular vein, and confirmed post-procedurally with chest X-rays. No cases of catheter malposition were observed among our patients.

All patients in our study underwent catheterization using the in-plane technique. None experienced bleeding, arterial injury, or nerve damage. Previous studies have shown that the use of ultrasound reduces these risks (10). The absence of such complications in our study supports these findings.

Applying pressure to the area where the subclavian vein is located is quite challenging. Therefore, accidental subclavian artery puncture poses a significant risk of bleeding. One of the primary advantages of real-time ultrasound guidance is the precise identification of the boundaries of the subclavian artery and vein, allowing for accurate needle placement within the subclavian vein lumen (11).

This approach helps prevent subclavian artery injury. The risk of thrombosis associated with central venous catheterization is reported to be 5%–10% in the literature. Risk factors include the catheter’s material (e.g., length, polyethylene composition, lack of flexibility), its use for parenteral nutrition, and prolonged catheter duration. Hyperosmolar solutions can cause chemical irritation and perforation of the vessel wall, leading to connective tissue damage and increased vascular permeability (12). In our study, thrombosis occurred in only one patient, who was receiving total parenteral nutrition.

We believe that, influenced by our publication and others, anesthesiologists working in both operating rooms and intensive care units will gain confidence in ultrasound-guided subclavian catheterization. With proper ultrasound training, physicians will be able to visualize the subclavian artery, subclavian vein, pleura, and lungs with ease. This enables real-time guidance of the needle and precise adjustments as needed. Once the needle enters the subclavian vein, a guidewire can be introduced, followed by the placement of a central venous catheter over the guidewire.

Another advantage of ultrasound use is the ability to assess for procedure-related pneumothorax post-intervention. Ultrasound is a highly sensitive and specific imaging modality for detecting pneumothorax. However, additional training for practitioners is necessary to ensure proficiency. Studies have reported an ultrasound sensitivity of 89% and a specificity of 96% for pneumothorax detection, which are remarkably high rates. In current practice, ultrasound is widely used as the first imaging modality in trauma patients and is commonly employed for pneumothorax and pleural effusion assessment. Nevertheless, imaging can be challenging in patients with morbid obesity, extensive subcutaneous emphysema, or large dressings (11, 13).

The presence of thrombosis can pose a significant challenge during the catheterization procedure. The use of ultrasound facilitates the detection of thrombi within the vessel, preventing unnecessary interventions in occluded veins. Moreover, ultrasound evaluation enables the identification of the most suitable anatomical entry site for the procedure. In general, compression tests are used to assess thrombosis in the jugular and femoral veins. However, due to the anatomical location of the subclavian vein, compression-based evaluation has limitations, as the vein is not easily compressible. Instead, the presence of hypoechoic, irregularly bordered structures within the vessel lumen may indicate thrombosis. In such cases, selecting an alternative site for the procedure is advisable, and ultrasound should be utilized to confirm its suitability for catheterization (11).

The literature indicates that training anesthesia trainees in ultrasound-guided catheterization is highly beneficial (1). In our study, confidence assessments conducted before and after training showed a significant increase in trainees’ confidence in independently performing catheterization. It should be noted that our study is retrospective, records were kept this way. We believe it would be better to conduct prospective, multicenter studies using our study as an example and administer a more objective trust survey to participants.

This study has certain limitations, including its retrospective design, relatively small sample size, and the exclusive inclusion of anesthesia trainees. Future studies involving larger patient populations and more diverse groups of practitioners are warranted to strengthen these findings.

## Conclusion

We believe that anesthesiologists and anesthesia trainees, who routinely use ultrasound and possess basic eye-hand coordination skills, can safely and effectively perform ultrasound-guided subclavian catheterization in the intensive care unit. The use of ultrasound in catheterization reduces the risk of complications, and we strongly advocate for its mandatory use in catheter placement procedures for critically ill patients.

## Data Availability

The original contributions presented in this study are included in this article/supplementary material, further inquiries can be directed to the corresponding author.
